# A modified device to place abomasal infusion lines for rumen-cannulated cattle

**DOI:** 10.3168/jdsc.2021-0201

**Published:** 2022-04-26

**Authors:** L.R. Rebelo, C. Lee

**Affiliations:** Department of Animal Sciences, Ohio Agricultural Research and Development Center, The Ohio State University, Wooster 44691

## Abstract

A previously developed abomasal infusion device was modified for easy and successful placement of infusion lines into the abomasum of dairy cows. Similar to the original device, the modified device consists of 2 pieces: an insertion tool and a delivery tool. Updates include streamlining both the insertion and delivery tools by slightly altering dimensions and design, then smoothing all rough edges. The primary changes include a parallel cut along the entire length of the insertion and delivery tools and encasement of the infusion line inside both tools, allowing for smoother insertion through the reticulum-omasum orifice and into the omasum and abomasum. Additionally, increasing the outside diameter of the delivery tool to reduce the gap between the delivery tool and insertion tool, increasing the length of the tools, and making a loop of cord attached to the insertion tool facilitate insertion and easy ejection of the flange into the abomasum. By using this modified device, placement of abomasal infusion lines (including flange) was more successful.

## Highlights

• Abomasal infusion is a critical technique to understand postruminal metabolism.
• The infusion device was modified for successful placement of the infusion line.
• The device was successful in placing the infusion line without reinsertion for 6 days.

## Graphical Abstract

**Figure:**
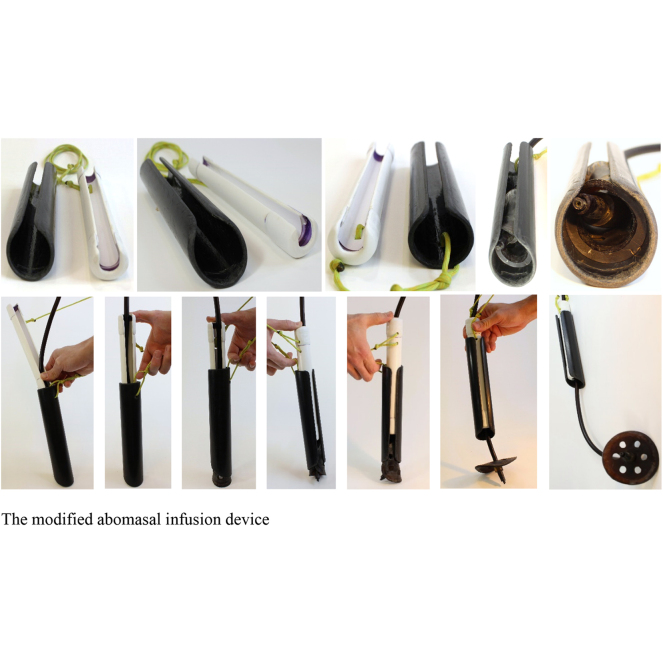
**Summary:** Investigations of postruminal metabolism provide fundamental information about postruminal nutrient metabolism, and a postruminal infusion technique allows such assessment. As a technique, a flange is used to anchor the end of the infusion line in the abomasum. The placement of an abomasum infusion line with a flange in large cows has been done with insertion and delivery tools. Although a previously developed device allowed successful placement of flanges, several difficulties were found. Therefore, we modified the device and found that the modified device is easier and less time consuming in placing an infusion line into the abomasum.

Abomasal infusion of nutrients and markers in cattle is a fundamental technique used to improve understanding of postruminal nutrient metabolism. Cannulating cattle in the abomasum (Abramson et al., 2002) or the duodenum (Titgemeyer et al., 1989) enables postruminal infusions; however, such cannulations have become uncommon because of the difficulty to maintain those animals and animal welfare concerns. Without the abomasal and duodenal cannulation, an infusion device made of 6-mm polyethylene tubing attached to a perforated 60-mL bottle was initially developed. The perforated bottle was anchored in the abomasum passing through the rumen cannula, reticulum-omasum, and omasum-abomasum orifices (Derrig et al., 1974; Spires et al., 1975). Modifications to the infusion device have been made, such as the addition of a plastic flange to the end of the line before the perforated plastic bottle (Oldick et al., 1997; Haubold et al., 2020; Rico et al., 2021) or the plastic flange without the bottle (Romo et al., 1996; Knowlton et al., 1998; Benson et al., 2001; Larsen and Kristensen, 2009). The plastic flange or bottle should be anchored appropriately in the abomasum for successful placement of infusion lines and infusion of nutrients. However, researchers with an average arm length often had difficulty reaching the abomasum to appropriately place the device. To facilitate the placement of the infusion line with a plastic flange without the need for the hand and forearm to enter the omasum, a device consisting of 2 polyvinyl chloride (**PVC**) pieces (insertion and delivery tool) was developed by Gressley et al. (2006).

After the device by Gressley et al. (2006), another device with a metal object was developed that is placed in the abomasum without the need for insertion and delivery tools and a plastic flange (Westreicher-Kristen and Susenbeth, 2017). The device consisted of a perforated stainless-steel object weighing 125 g and an infusion line attached to the object. According to Westreicher-Kristen and Susenbeth (2017), placement of the infusion line is even less invasive than the aforementioned technique by Gressley et al. (2006) because when the metal object is placed in the omasum, it can reach the abomasum by its weight and gravity. The technique was successful in maintaining the metal object and infusion line in the abomasum for days in studies with Jersey cows (Westreicher-Kristen and Susenbeth, 2017). However, our preliminary study with a large Holstein cow (722 kg of BW) revealed that the metal object remained inside the abomasum only for a few hours, resulting in the object and tubing being in the rumen. Furthermore, there was difficulty with the large lactating cow to confirm the placement of the metal object in the abomasum when the procedure from Westreicher-Kristen and Susenbeth (2017) was followed. While troubleshooting problems with the stainless-steel object attached to the infusion tubing, we found that decreasing the infusion tubing length from 2.5 m (length recommended by Westreicher-Kristen and Susenbeth, 2017) to 1.5 m doubled the time that the metal object remained in the abomasum (from about 2 to 4 h). However, this technique was deemed unsuccessful when used for multiple days of infusion in large Holstein lactating cows.

After the failure of placing the infusion line with the technique by Westreicher-Kristen and Susenbeth (2017), we decided to use the tools developed by Gressley et al. (2006) in an abomasal infusion experiment. With a 1.5-m infusion line, the metal object was replaced with a plastic flange that was used in Gressley et al. (2006) with small modifications where the flange used had a diameter of 10 cm and 5 mm in height and a washer was placed only on one side of the flange (Figure 2G). In addition, the infusion tubing type and diameter was similar to the one used in Westreicher-Kristen and Susenbeth (2017). In this experiment, 4 Holstein lactating cows (659 ± 42 kg of BW) were used in a 4 × 4 Latin square design. Although we were able to conduct the infusion using this technique during the experiment, there were difficulties placing and fully maintaining the infusion line (i.e., flange) in the abomasum for 6 d in each period. These difficulties occurred due to inappropriate anchor of the flange in the abomasum. Therefore, when the placement of the flange for individual cows was checked twice a day during the 6-d infusion, the flange was often found in the omasum (i.e., close to the reticulum-omasum orifice). In such cases, the flange was immediately reinserted with the device. All the cows received the reinsertion of the device at least more than once during the 6-d infusion periods.

The following are the specific obstacles found when the device by Gressley et al. (2006) was used to place infusion lines. Primarily, the 45°-angled end of the insertion tool has 2 parallel cuts about halfway down the length of the tool. This produces 2 half-cylinder shapes that may result in weak sidewalls depending on the PVC material used, thereby potentially contributing to an outward expansion of the tool when the flange is placed. Furthermore, the design of the device requires the infusion line to be placed exterior to the insertion tool when the flange is placed. The combination of the increased breadth and irregularities as the infusion line slides around the insertion tool causes resistance when passing through the reticulum-omasum orifice, omasum, and omasum-abomasum orifice. Finally, a probable reason for having the flange pulled into the rumen several times throughout the study was that the insertion tool was not long enough to fully pass the omasum-abomasum orifice, causing incomplete anchoring with the flange.

After the experiment, modifications were made to the device developed by Gressley et al. (2006). The modifications in detail with dimensions are depicted in Figure 1. Briefly, the modified insertion and delivery tool were made out of PVC pipes as described in Gressley et al. (2006). The insertion tool included a single plane bias cut (approximately 45° angle) on one of the ends of a PVC pipe (Figure 1A) as in Gressley et al. (2006). From the flat end to leading edge of the 45°-angled bevel, the maximum length is 24.5 cm and the minimum length of the tool is 22 cm. Along this short length, a parallel cut of 1.2 cm width exists to allow placement of the infusion line inside the tool. The delivery tool consists of 23 cm length of a 3.2-cm o.d. PVC pipe with PVC-end caps (Figure 1B). Two 3.2-cm i.d. PVC caps were affixed to both ends of the PVC delivery tool such that the total length of the tool is 24.5 cm. Because the capped delivery tool o.d. was larger than the i.d. of the insertion tool, the cap o.d. was reduced to 3.6 cm with a disc sander, bench grinder, and sandpaper. With the o.d. of 3.6 cm, rather than 3.3 cm as in Gressley et al. (2006), the gap between the delivery and insertion tool is smaller. Along the entire length, the delivery tool has a parallel cut of 1.3 cm width, which allows the infusion line to be placed inside the tool. One hole (0.5 cm of diameter) was drilled on the flat end of the insertion tool (3 cm from the end; Figure 1A), and 2 holes (0.5 cm of diameter) were drilled at 3.3 and 5 cm from one of the edges of the delivery tool (Figure 1B). The holes are used to tie nylon cords with a loop (Figure 2D). The nylon cord tied both tools together and the length of the cord was about 1 m long. All the edges of the tools were smoothed with sandpaper.

**Figure 1:**
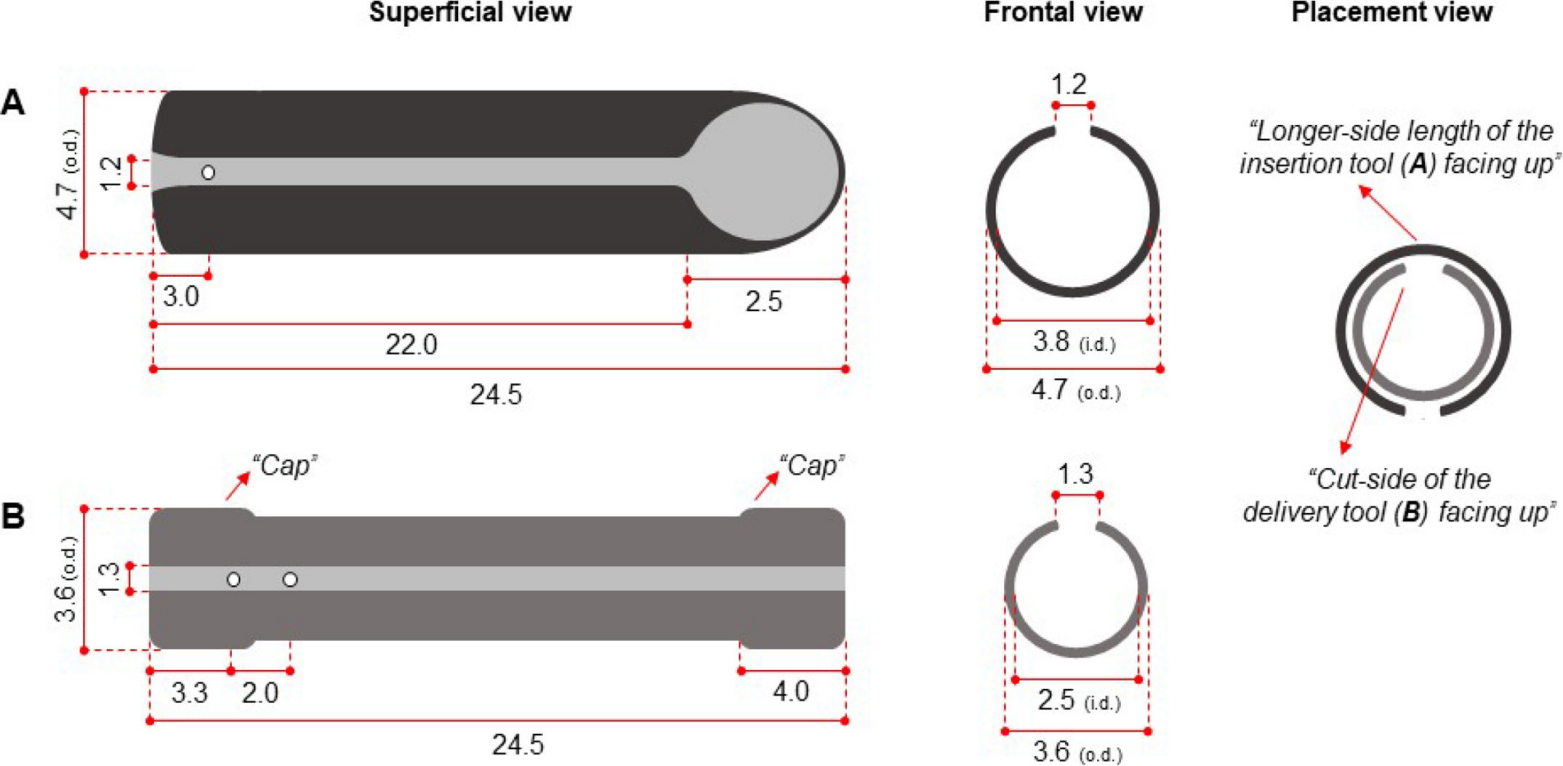
Measurements of the insertion tool (A) and delivery tool (B). Placement view refers to the recommended position of which both tools should be inserted through the reticulum-omasum orifice, omasum, and omasum-abomasum orifice. All measurements are given in centimeters. o.d. = outside diameter; i.d. = inside diameter.

**Figure 2:**
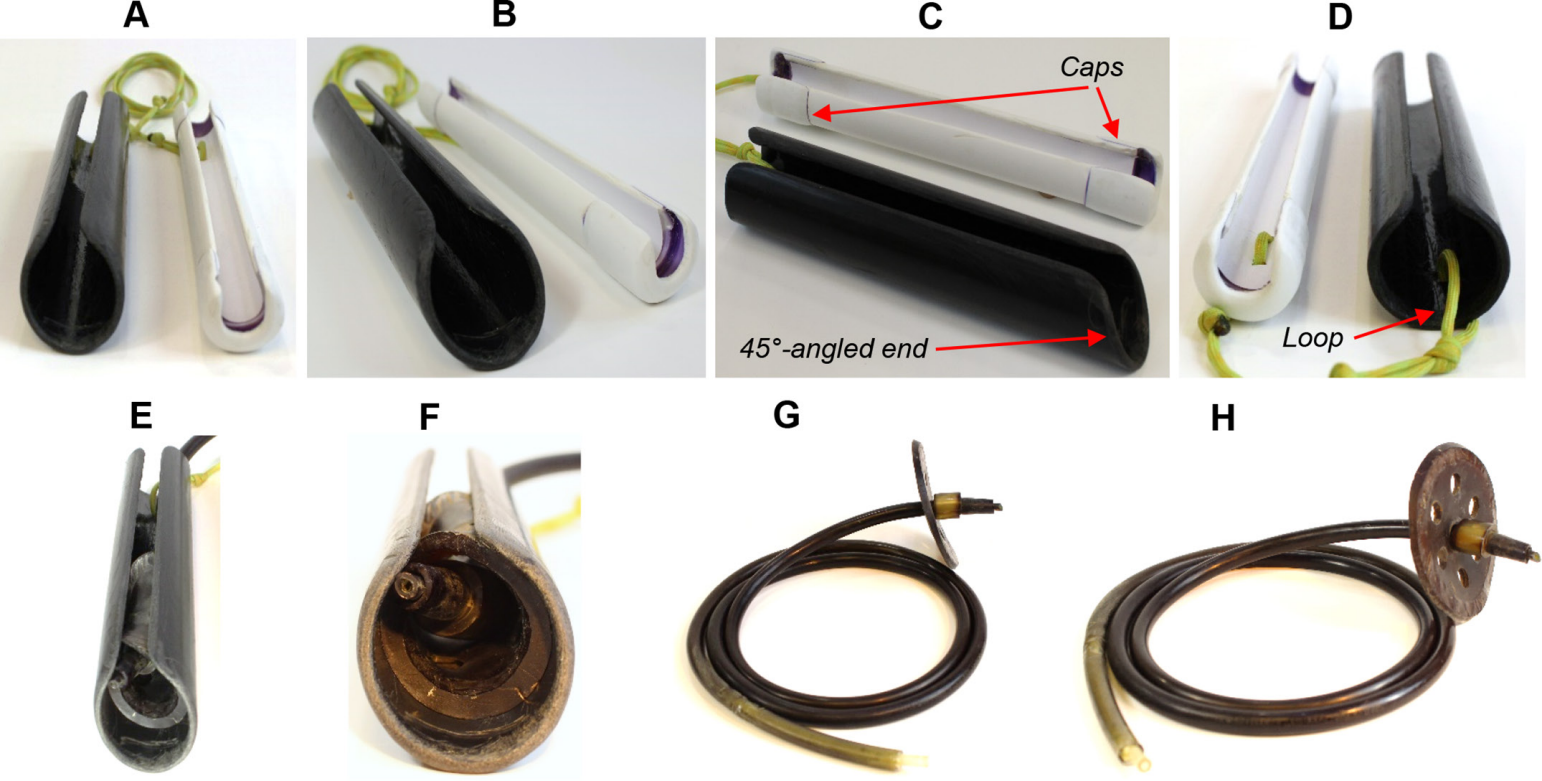
The view of the updated insertion (black) and delivery (white) tool: front (A), front close-up (B), side (C), back (D), front with flange (E), and close-up of front with flange (F), infusion line with a flange (G), and the flange with 6 peripheral holes (H).

Removing one of the parallel cuts on the insertion tool from Gressley et al. (2006) eliminated the resultant expansion when the flange is placed in the insertion tool. The bias cut on the updated insertion tool also allowed a smoother passage of the tool through the reticulum-omasum orifice and the omasum compared with the tool from Gressley et al. (2006). Furthermore, with the infusion line inside the insertion tool (compared with being outside as in Gressley et al., 2006), it eliminated the asymmetry and resistance caused by the additional breadth from the line, thus making insertion of the insertion tool through the reticulum-omasum orifice, omasum, and omasum-abomasum orifice much smoother. The single cut made along the extent of both tools allows the infusion line to remain in the insertion tool during insertion of the tool into the abomasum (Figure 3). Increasing the length of both tools (1.5 cm) seems appropriate to place the flange in the abomasum especially for mature Holstein cows. Addition of PVC caps to the delivery tool provides a larger surface area for pushing the tool with thumb or index finger, increasing leverage, thus requiring less strength to eject the flange. Furthermore, the increased o.d. of the delivery tool with caps made the tighter fit between the insertion and delivery tool, which prevents jams that often occurs between the delivery tool and the flange in the insertion tool when the delivery tool is pushed to release the flange. The original device (Gressley et al., 2006) has a 7-mm gap between the delivery and insertion tool and we experienced jams of the delivery tool with the flange (without oils or lubricants applied). After inserting the insertion tool into the abomasum, Gressley et al. (2006) recommended wrapping the cord from the delivery tool around the palm of the hand to add torque to eject the flange from the delivery tool. We also found that the time taken to complete the procedure was reduced with a loop of the cord tied to the tools.

**Figure 3:**
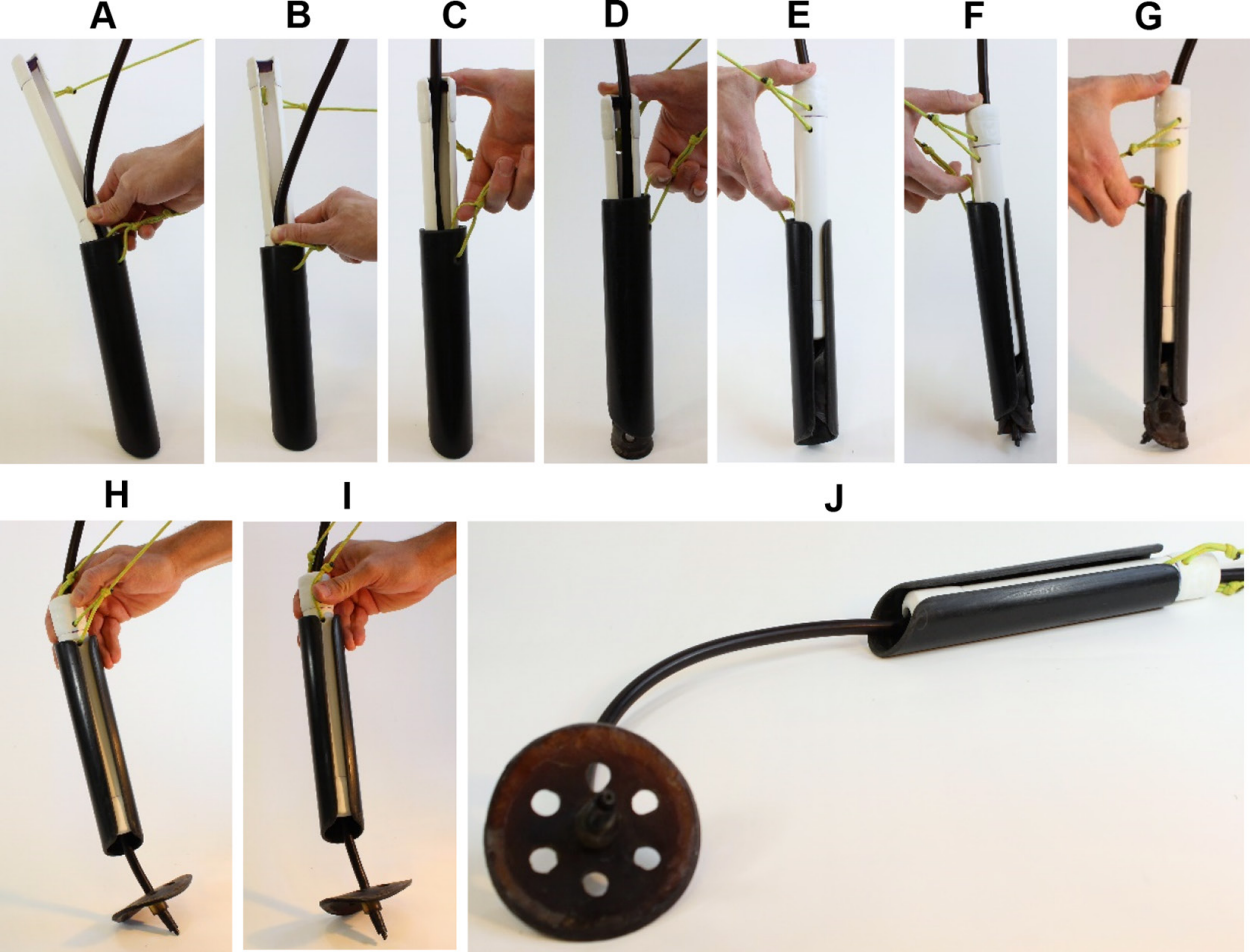
Step-by-step (A–J) images of flange ejection using the updated insertion tool (black) and delivery tool (white).

The following is the procedure to use the modified device. As the flange usually hardens when stored at room temperature, the flange is left for 5 to 10 min in hot water to soften immediately before use. Although breeding lubricant (Gressley et al., 2006) or vegetable oil can be used to make flange ejection smooth, this was not necessary with the modified device. The infusion line attached to the flange was placed in the insertion device and the softened flange was folded and placed into the insertion tool (Figure 2E–F). Although Westreicher-Kristen and Susenbeth (2017) recommended using the left arm for the procedure, we found that use of the right arm as in Gressley et al. (2006) was more convenient. Initially, the insertion tool is first introduced into the reticulum with care to avoid large feed particles entering the insertion tool. Then, while holding the end of the insertion tool (the 45°-angled end where the flange is placed) with the thumb and index finger, the fifth finger is used to find the reticulum-omasum orifice. When the orifice is found, the 45°-angled end of the insertion tool is slowly inserted into the omasum through the orifice. The best way to pass the insertion through the orifice is to have the longer length side of the tool facing up (i.e., dorsally to the cow; see Figure 1 for details) with the shorter side length (due to the bias cut) facing down (i.e., ventrally to the cow). After inserting the tool through the orifice, the step-by-step procedure can be followed as described in Gressley et al. (2006).

In our experiment, placement of the infusion line with the device was successful before feeding. We also found that it was successful 1 to 2 h after feeding (when the rumen was not completely full) and felt that the reticulum-omasum orifice was loosened after feeding (compared with before feeding), which made passing the tools through the orifice and omasum easier. We speculate that loosening of the orifice tends to happen after feeding to allow greater passage. A sudden flow coming from the abomasum through the insertion tool is a good indicator that the end of the insertion tool reached the abomasum as described in Gressley et al. (2006). The infusion line remains inside the insertion tool throughout the whole procedure. After the insertion tool is almost entirely inserted into the omasum, the right arm is removed from the rumen to obtain the delivery tool. If large feed particles are present inside the insertion tool, they can be removed using fingers. The delivery tool with the cut side facing up (i.e., dorsally; Figure 1) is positioned right before the insertion tool and then the infusion line is placed inside the delivery tool as shown in Figure 3A–B. Sequentially, the index finger is hooked in the cord loop on the insertion tool and the thumb is used to gently push the delivery tool through the insertion tool (Figure 3C–G). When the delivery tool reaches the flange inside the insertion tool, about a half of the delivery tool should be placed inside the insertion tool (Figure 3E). If the insertion tool is pulled out from the omasum during inserting the delivery tool, then a gentle pressure is needed to push the insertion tool (the delivery tool is still inside the insertion tool) toward the abomasum until the insertion tool almost fully passes the reticulum-omasum orifice (i.e., only 1 to 2 cm of the tool end is in reticulum). When the insertion tool almost fully passes the reticulum-omasum orifice, this means that the 45°-angled end of the tool passes the omasum-abomasum orifice. Then, the flange is released into the abomasum by further pushing the delivery tool (Figure 3C–G). If the device does not enter through the omasum-abomasum orifice, the insertion tool (with the delivery tool still inside) should be pulled backward 2 to 4 cm, and then gently pushed again toward the abomasum direction as described in Gressley et al. (2006). If the end of the insertion tool is correctly placed inside the abomasum, the release of the flange should not require much strength (when pushing the delivery tool). After the flange is fully released (Figure 3H–I), both the insertion and delivery tool are pulled together along the infusion line (Figure 3J) and removed from the rumen. The right arm is then reinserted into the reticulum to check the position of the flange. The index finger can be used to check if the flange is touched inside the omasum. If the flange is not touchable, it means that the flange was positioned inside the abomasum properly in most cases. However, because we observed the flange pulled back into the omasum when we used the device from Gressley et al. (2006), although the flange was not touchable when placed, we recommend checking the position of the flange 2 or 3 h after placement and daily during the experiment.

Experiments that require abomasal infusion are usually expensive and highly time-consuming, and when infusion lines are incorrectly placed, it results in a large variation in data and potentially inaccurate results. This modified device posed no detrimental health effects to the cows. We also observed no effects on DMI and milk production during the day of placement of the infusion line, nor in subsequent days thereafter. Because of the shape and size of the modified device, the placement of the flange in the abomasum was smoother and quicker compared with the device from Gressley et al. (2006). Additionally, we found results opposite to Westreicher-Kristen and Susenbeth (2017) in terms of the length of the infusion line. Westreicher-Kristen and Susenbeth (2017) recommended an infusion line 2.5 m in length. With the flange-type infusion device, we found that the line with this length often became tangled with rumen content and the flange was pulled out of the abomasum. When tubing was shortened to 1.5 m, it substantially decreased the number of times the line was tangled in the rumen. It must be noted that the length of the line may need adjustment according to animal size and diet type. The length of 1.5 m was successful for modern Holstein dairy cows (primiparous and multiparous) fed a typical Midwest diet (50 to 60% forage and 40 to 50% concentrate).

The modified insertion and delivery tools with the tubing length of 1.5 m was used in an infusion experiment with a 4 × 4 Latin square design, where 4 rumen-cannulated Holstein cows received continuous abomasal infusion for 6 d (totaling continuous 144 h per cow) during each period. Placement of the flanges was checked immediately after insertion and twice daily for all cows during 6 d of infusion in each period. In this experiment, reinsertion of the flanges was not necessary, and we did not observe any flanges that were pulled from the abomasum for 6 d of infusion. All procedures were previously approved by the Institutional Animal Care and Use Committee of The Ohio State University.
